# Analysis of short-term heart rate and diastolic period variability using a refined fuzzy entropy method

**DOI:** 10.1186/s12938-015-0063-z

**Published:** 2015-07-01

**Authors:** Lizhen Ji, Peng Li, Ke Li, Xinpei Wang, Changchun Liu

**Affiliations:** School of Control Science and Engineering, Shandong University, 17923 Jingshi Road, Jinan, 250061 People’s Republic of China

**Keywords:** Heart rate variability, Diastolic period variability, Coronary artery stenosis, Refined fuzzy entropy, Fuzzy entropy, Sample entropy

## Abstract

**Background:**

Heart rate variability (HRV) has been widely used in the non-invasive evaluation of cardiovascular function. Recent studies have also attached great importance to the cardiac diastolic period variability (DPV) examination. Short-term variability measurement (e.g., 5 min) has drawn increasing attention in clinical practice, since it is able to provide almost immediate measurement results and enables the real-time monitoring of cardiovascular function. However, it is still a contemporary challenge to robustly estimate the HRV and DPV parameters based on short-term recordings.

**Methods:**

In this study, a refined fuzzy entropy (rFuzzyEn) was developed by substituting a piecewise fuzzy membership function for the Gaussian function in conventional fuzzy entropy (FuzzyEn) measure. Its stability and robustness against additive noise compared with sample entropy (SampEn) and FuzzyEn, were examined by two well-accepted simulation models—the $1/f^{\alpha }$ noise and the Logistic attractor. The rFuzzyEn was further applied to evaluate clinical short-term (5 min) HRV and DPV of the patients with coronary artery stenosis and healthy volunteers.

**Results:**

Simulation results showed smaller fluctuations in the rFuzzyEn than in SampEn and FuzzyEn values when the data length was decreasing. Besides, rFuzzyEn could distinguish the simulation models with different amount of additive noise even when the percentage of additive noise reached 60%, but neither SampEn nor FuzzyEn showed comparable performance. Clinical HRV analysis did not indicate any significant differences between the patients with coronary artery disease and the healthy volunteers in all the three mentioned entropy measures (all *p* > 0.20). But clinical DPV analysis showed that the patient group had a significantly higher rFuzzyEn (*p* < 0.01) than the healthy group. However, no or less significant difference was observed between the two groups in either SampEn (*p* = 0.14) or FuzzyEn (*p* = 0.05).

**Conclusions:**

Our proposed rFuzzyEn outperformed conventional SampEn and FuzzyEn in terms of both stability and robustness against additive noise, particularly when the data set was relatively short. Analysis of DPV using rFuzzyEn may provide more valuable information to assess the cardiovascular states than the other entropy measures and has a potential for clinical application.

## Background

Cardiovascular diseases (CVDs) have become the leading cause of death over the world [1, 2]. One of the most commonly encountered types of CVDs is coronary heart disease [3], which is typically characterized by coronary artery stenosis. At most cases, the deteriorated cardiovascular function would have become irreversible after being diagnosed. It is thus meaningful to detect the CVDs at an earlier stage before the occurrence of common clinical symptom and organic lesion (usually named as a subclinical phase), ideally in a non-invasive and non-destructive way [4].

Most CVDs are theorized to bring disruption in autonomic control [2, 5]. Besides, the heart rate variability (HRV) has been proved to be capable of probing into the autonomic regulation. Thus, researchers have developed the HRV analysis to evaluate the cardiovascular functioning indirectly [6–8]. The HRV refers to tiny fluctuations between normal sinus heartbeats which are often represented by the consecutive RR intervals from the ECG data. Previous results have already linked the HRV alterations to CVDs [9–11]. Moreover, one cardiac cycle comprises of two complementary electromechanical periods, viz. cardiac systolic and diastolic periods. Both periods show variability and exhibit certain coupling with the heart period [12]. We have shown that the changes of diastolic period variability (DPV) in healthy elderly subjects [13] and heart failure patients [14] are different from those of HRV. Thus DPV may contribute more information beyond the HRV to evaluation of coronary artery stenosis.

Clinically, there is a growing need for short-term (usually about 5 min) HRV and DPV examinations, particularly in a scenario of point-of-care diagnosis or mobile healthcare service etc. [15, 16]. However, the common time- and frequency-domain methods lack reliability and reproducibility in short-term HRV/DPV analysis due to its non-stationary nature [6]. Traditional nonlinear measures, such as fractal dimension, correlation dimension, and Lyapunov exponents, require sufficient data length (typically should be long enough for trajectory reconstruction) [17, 18]. Therefore, they are also inapplicable in short-term HRV/DPV examinations.

Entropy measures, such as sample entropy (SampEn), have showed potential in reliable short-term analysis [19]. Within a time-series of length *N*, if two vectors of certain length *m* are close to each other (within a tolerance *r*), SampEn measures the likelihood that the two vectors will remain close on the next incremental comparison. It is however limited by sensitivity to both *N* and *r* [20]. To attenuate the parameter effects, Chen et al. [21] and Xie et al. [22] introduced independently the fuzzy logic to the classification procedure and defined as fuzzy entropy (FuzzyEn) measure. Nevertheless, switched FuzzyEn values have also been observed under different values of *r* [23]. Its performance in short-term series thus requires further elucidation. Most recently, we have shown that, in a multivariate analysis framework, the fuzzy membership function (e.g., the Gaussian function) should be a potential influencing factor to the statistical performance of FuzzyEn. After substituting a piecewise function for the Gaussian function, results have shown significantly improved stability [24].

Therefore, in this study, we firstly developed a new FuzzyEn measure based on the piecewise fuzzy membership function. The performance of our proposed new FuzzyEn—refined FuzzyEn (rFuzzyEn)—was then tested on synthetic data and the rFuzzyEn was finally applied to analyse the 5-min human HRV and DPV series for both the patients with coronary artery stenosis and healthy volunteers.

## Methods

### Algorithms of SampEn and FuzzyEn

For a normalized (with null mean and unit standard deviation) length-*N* series $\left\{ {x_{i} ,i = 1,2, \ldots ,N} \right\}$, given the input parameter *m* (embedding dimension), form vector ${\mathbf{X}}_{i}^{\left( m \right)}$ as ${\mathbf{X}}_{i}^{\left( m \right)} = \left[ {x_{i} ,x_{i + 1} , \ldots ,x_{i + m - 1} } \right]$ ($i = 1,2, \ldots ,N - m$). Define the distance between two vectors ${\mathbf{X}}_{i}^{\left( m \right)}$ and ${\mathbf{X}}_{j}^{\left( m \right)}$ by

1 $$ d_{ij}^{\left( m \right)} = \left. {\hbox{max} \left( {\left| {x_{i + k} - x_{j + k} } \right|} \right)} \right|_{k = 0}^{m - 1} , $$

where $i,j = 1,2, \ldots ,N - m$. For each ${\mathbf{X}}_{i}^{\left( m \right)}$, define the average number of ${\mathbf{X}}_{j}^{\left( m \right)}$ ($j = 1,2, \ldots ,N - m \cap j \ne i$) which is similar to ${\mathbf{X}}_{i}^{\left( m \right)}$ by

2 $$ B_{i}^{\left( m \right)} \left( r \right) = \frac{{\sum\limits_{j = 1,j \ne i}^{N - m} {\Theta \left( {r - d_{ij}^{\left( m \right)} } \right)} }}{N - m - 1}, $$

where *r* is the threshold parameter, $\Theta ( \cdot )$ indicates the Heaviside function. Then, compute the mean of $B_{i}^{\left( m \right)} \left( r \right)$ by

3 $$ B^{\left( m \right)} \left( r \right) = \frac{{\sum\limits_{i = 1}^{N - m} {B_{i}^{\left( m \right)} \left( r \right)} }}{N - m}. $$

Similarly, define $B_{i}^{{\left( {m + 1} \right)}} \left( r \right)$ as the average number of ${\mathbf{X}}_{j}^{{\left( {m + 1} \right)}}$ which is similar to ${\mathbf{X}}_{i}^{{\left( {m + 1} \right)}}$, and compute its mean $B^{{\left( {m + 1} \right)}} \left( r \right)$, accordingly [19]. Finally, the SampEn can be estimated by

4 $$ SampEn\left( {m,r,N} \right) = - \ln \frac{{B^{{\left( {m + 1} \right)}} \left( r \right)}}{{B^{\left( m \right)} \left( r \right)}}. $$

For FuzzyEn, the Heaviside function in (2) is substituted by a Gaussian function

5 $$ A\left( {d,r} \right) = e^{{ - \ln \left( 2 \right)\left( {d/r} \right)^{2} }} , $$

more details are available in [25, 26].

### Refined FuzzyEn algorithm

The Gaussian function shown in (5) declines smoothly with increasing *d*. It is different from the Heaviside function which provides a rigid boundary (either 1 or 0) to scale the similarity degree of two vectors. With the Heaviside function, a slight variation of *d* around *r* can lead to abrupt changes in calculation of similarity degree. But this effect can be largely attenuated in FuzzyEn.

However, damping in Gaussian function appears right after *d* > 0. But mostly, a small *d* may not necessarily indicate a real difference between two vectors. Recurring to the small threshold *r* in SampEn, it has been initially introduced to distinguish between the similar and dissimilar vectors. Any one which is within *r* of a certain vector is ranked to be similar, whereas the distance *d* ($0 \le d \le r$) between them is considered to be artefact brought about by possible sampling errors or disturbance [24].

We have introduced a transient plateau at the very beginning of the Gaussian function shown in (5) and developed accordingly a new piecewise fuzzy membership function

6 $$ A\left( {d,r} \right) = \left\{ {\begin{array}{*{20}c} {1,} & {0 \le d < r} \\ {e^{{ - \ln \left( 2 \right)\left( {\frac{d - r}{r}} \right)^{2} }} ,} & {d \ge r} \\ \end{array} } \right. , $$

which can significantly increase stability and consistency of the multivariate fuzzy entropy [24]. We thus also expect increased performances of FuzzyEn with this new fuzzy membership function. By substituting (6) for the Heaviside function in (2), we here define the rFuzzyEn, and will then make further simulation tests on its stability and robustness to additive noise in following sections. Subsequently, the so-defined rFuzzyEn will be applied to short-term HRV and DPV analysis.

### Simulation study

#### Simulation models

Two well-studied simulation models with inherently stochastic and chaotic natures, respectively, were employed in this study. The first one was the power law distribution noise—the $1/f^{\alpha }$ noise model, which corresponds to white noise when $\alpha = 0$, pink noise when $\alpha = 1$, and finally Brownian motion when $\alpha = 2$. The pink noise ($1/f$ noise) has a long-range memory effect and is commonly applied to represent the heartbeat rhythms [27]. Another model was the Logistic attractor $x\left( {n + 1} \right) = \mu \times x\left( n \right) \times \left( {1 - x\left( n \right)} \right)$ with $\mu = 3.5$ to represent a limit cycle (periodic series), and with $\mu = 4.0$ to represent a chaotic regime. This model has also been widely applied to characterize the behaviour of physiological systems [28].

#### Stability examination

The $1/f^{\alpha }$ noise models with $\alpha = 0$, 1, and 2, were applied in this test, respectively. The Logistic attractors were not used here because the attractors usually show different regimes with different $\mu$ values even in very small data set, and thus, even traditional measures can well differentiate between each other.

We monitored the conventional SampEn, FuzzyEn and the rFuzzyEn as functions of data length *N*, which was set at 100–2,000 logarithmically in our simulation. All three $1/f^{\alpha }$ noise models were produced independently for empirical 20 times for each value of *N* to eliminate random factors [29]. The standard deviation of the 20 realizations was used as a measure of stability.

#### Examination of the robustness against additive noise

The Logistic attractors with $\mu = 3.5$ and 4.0, respectively, were applied in this test, since they are significantly different in their complexity levels, and can be distinguished from each other simply by all the three entropy measures. However, the intrinsic sequence structures will be contaminated by additive noise and the discrimination task will become relatively hard with the presence of noise.

To investigate the robustness against additive noise of SampEn, FuzzyEn, and the rFuzzyEn, we monitored their performances with the percentage of the additive noise. A length *N* = 300 was applied in this test, since this is approximately the average data length for 5 min HRV/DPV series. The additive noise was modelled by independently and identically distributed random noise and its percentage was set from 10 to 60% with a step of 5%. Both Logistic attractors were performed empirical 20 times independently for each value of the noise percentage to eliminate random factors [29]. The entropy measure with better robustness against additive noise could distinguish the two Logistic attractors even with relatively large noise percentage.

### Experimental study

#### Subjects

Thirty healthy volunteers (aged between 44 and 72, 13 males and 17 females) and 28 patients with coronary artery stenosis (aged between 42 and 73, 11 males and 17 females, *p* = 0.43 and 0.80, respectively) participated in the experiment. Health status of all volunteers was confirmed by questionnaires on their medical history and routine ECG and echocardiography examinations. Those who have undergone percutaneous coronary intervention or coronary artery bypass surgery were excluded prior to participation. All patients were confirmed by coronary angiography, and had at least one main coronary branch stenosed for over 50%. Routine ECG and echocardiography examinations were also implemented and no subject had frequent ectopic beats and left ventricular ejection fraction less than 50%. The study obtained full approval from the Institutional Review Board of Shandong Provincial Qianfoshan Hospital, and informed consent was required for each subject before participation. Table 1 shows their basic characteristics.

**Table 1:** Basic characteristics of the participants

Variables	Healthy volunteers	Patients with coronary artery stenosis	*p*
No. (m/f)	30 (13/17)	28 (11/17)	0.80
Age (years)	57.0 ± 7.4	58.7 ± 8.4	0.43
Height (cm)	165.8 ± 7.9	165.9 ± 7.5	0.94
Weight (kg)	63.9 ± 10.5	67.5 ± 8.7	0.13
BMI (kg/m^2^)	23.2 ± 2.5	24.1 ± 2.4	0.23
SBP (mmHg)	117 ± 10	121 ± 8	0.13
DBP (mmHg)	76 ± 5	78 ± 9	0.30

Data are expressed as number (male/female) or mean ± standard deviation (SD).

#### Protocol

Measurements were undertaken in a quiet and temperature controlled (25 ± 3°C) room at Shandong Provincial Qianfoshan Hospital, Shandong University, by a Cardiovascular Function Detection device (CV FD-II) produced by Huiyironggong Tech. Co., Ltd., Jinan, China. Before the formal signal recording, each subject lay supine on a measurement bed for a 10-min rest to allow cardiovascular system stabilization. ECG electrodes were attached to the right wrist and the right and left ankles to acquire a standard limb lead-II ECG. A photoelectric sensor was attached to the left forefinger tip to acquire fingertip photoplethysmography (PPG) waves. Subjects were told to breathe regularly and gently during the measurement.

**Figure 1:**
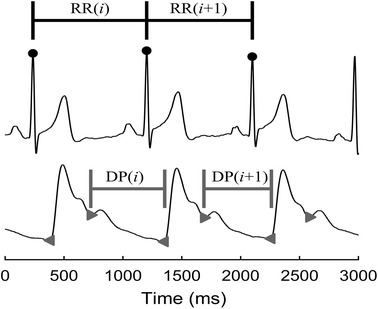
Construction of RR and DP intervals from simultaneously recorded ECG and PPG signals. The *upper waveform* represents the ECG signal, with the R-wave peaks marked by *dots*. The *bottom one* shows PPG signal, with the dicrotic notches and systolic feet marked by *triangles*.

For each subject, the ECG and PPG signals were synchronously recorded at a sampling frequency of 1 kHz for 5 min. R-wave peaks of ECG signals were extracted by a template-matching procedure [30]. The raw HRV series were constructed by consecutive R–R intervals. The systolic feet and dicrotic notches were detected from PPG by the first-order differential signals [31]. DPV series were constructed by intervals between the dicrotic notches and the following systolic feet. Anomalous intervals due to ectopic beats or poor signal quality were visually identified and removed in both the HRV and DPV series, simultaneously (no subject with more than 10% anomalous intervals was screened). Figure 1 represents the construction approach of RR and diastolic period (DP) intervals.

#### Time/frequency-domain analysis

Standard deviation (SD) of the raw HRV or DPV series was applied as the time-domain feature. Prior to frequency-domain analyses, the raw HRV and DPV series were evenly resampled with a sampling frequency of 4 Hz by spline interpolation. Their corresponding power spectral density (psd) was finally performed by the Burg’s method with 16th order. For both HRV and DPV, the psd was integrated in two frequency bands—from 0.04 to 0.15 Hz to obtain the power of low-frequency (LF) band and from 0.15 to 0.4 Hz the power of high-frequency (HF) band.

#### Entropy analysis

We calculated the rFuzzyEn measure of HRV and DPV, respectively, in the two groups. For comparison purposes, the conventional SampEn and FuzzyEn analyses were also performed. Note that in all computations of entropy, the raw HRV and DPV series without evenly resampling were used. The threshold value *r* is usually recommended to choose between 0.1 × *sd* and 0.25 × *sd* [32] (*sd* indicates the standard deviation of the under-analysed series), and Costa chose 0.15 × *sd* in analysis of biological signals by multiscale entropy [33]. Here, the *r* value for all three entropy measures was set at 0.15 × *sd*. The embedding dimension *m* was set at 2 [20].

### Statistical analysis

Normal distribution of the corresponding time-domain and nonlinear features of HRV or DPV was confirmed by the Kolmogorov–Smirnov test. Then the aforementioned indices were compared between healthy volunteers and patients with coronary artery stenosis by the student *t* test. Frequency-domain indices (LF and HF) in this study did not follow a normal distribution, which were thus compared between groups by the nonparametric Mann–Whitney *U* test. A statistical significance was accepted at *p* < 0.05. All statistical analyses were performed with SPSS (Ver. 20, IBM, USA).

## Results

### Simulation results

#### Stability

**Figure 2:**
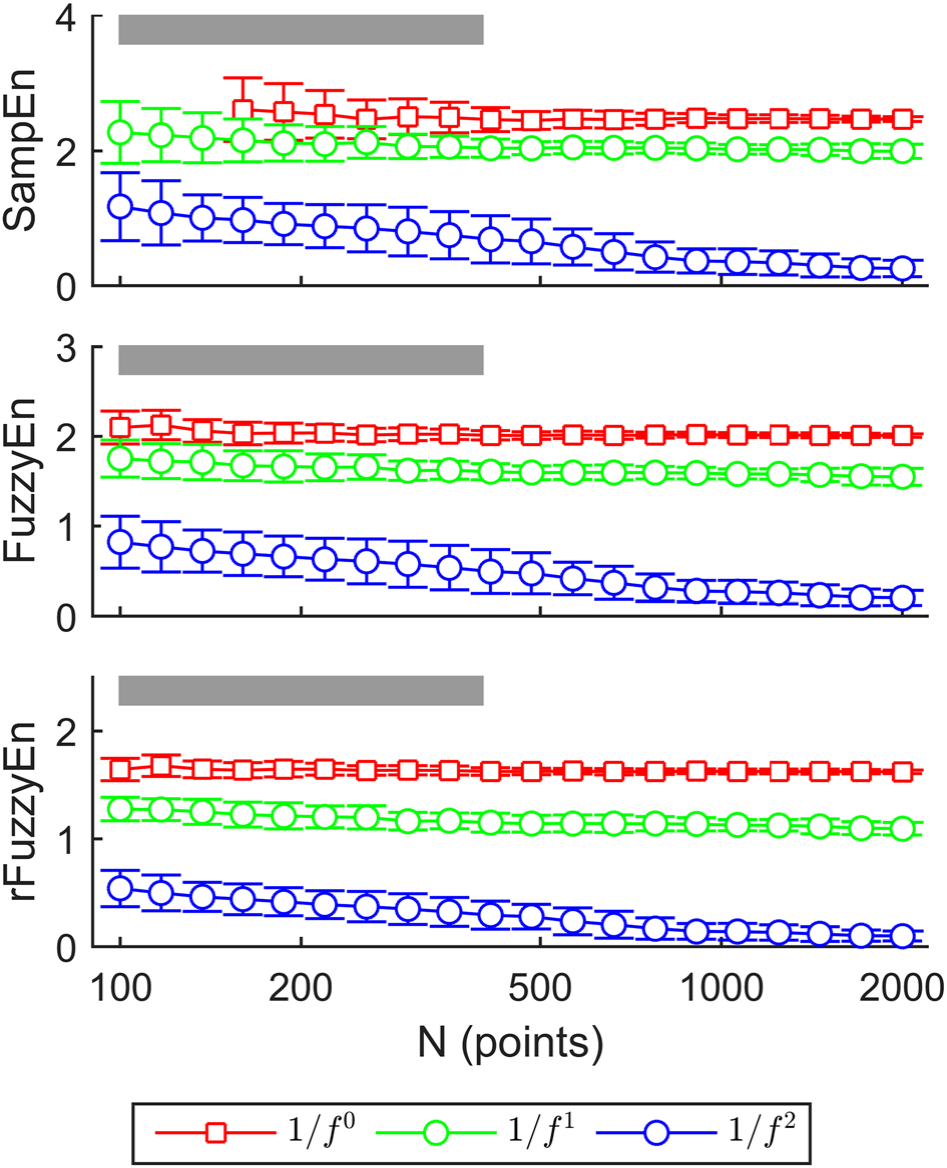
SampEn, FuzzyEn, and rFuzzyEn results of simulated $1/f^{\alpha }$ models with different data length. *Gray bar* indicates the lengths that cannot support good performance of SampEn and FuzzyEn, but can still for rFuzzyEn. The abscissa is in logarithmic scale for better visualization.

Figure 2 shows the SampEn, FuzzyEn, and rFuzzyEn as functions of data length *N*. In general, all three measures performed well when the length *N* > 1,000 points. For the $1/f^{2}$ and $1/f$ noise models, the standard deviations of SampEn and FuzzyEn were larger than those of rFuzzyEn. And for the $1/f^{0}$ noise, larger standard deviations were observed in *N* < 1,000 for SampEn, and in *N* < 500 for FuzzyEn, indicated by the gradually enlarged error bars in Figure 2. The SampEn of $1/f^{0}$ overlapped that of $1/f$ when *N* was no more than 500, and the FuzzyEn of $1/f^{0}$ overlapped that of $1/f$ when *N* was around 200. Invalid values were found in the SampEn of $1/f^{0}$ once the data length dropped to less than 200. By contrast, the rFuzzyEn showed excellent performance not only for large data set, but for short series, its performance was still acceptable (see the range marked by gray bar in Figure 2). Besides, the rFuzzyEn was still available to differentiate between the $1/f^{0}$ and $1/f$ models even if *N* reduced to as small as 100 data points.

### Robustness against additive noise

**Figure 3:**
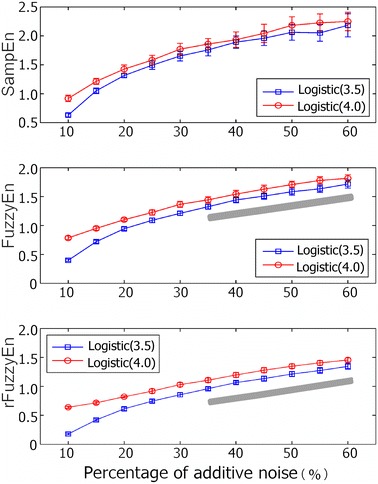
SampEn, FuzzyEn, and rFuzzyEn results of simulated Logistic attractors with different percentage of additive noise. *Gray bar* indicates the percentage of the additive noise that cannot support good performance of FuzzyEn, but can still for rFuzzyEn.

Figure 3 shows the SampEn, FuzzyEn, and rFuzzyEn as functions of additive noise percentage. All three measures performed well when the percentage of additive noise equals to 10 and 15%. SampEn of the Logistic attractors with $\mu = 4.0$ overlapped that of $\mu = 3.5$ when the noise percentage increased to 20%. The results showed a better performance with FuzzyEn than with SampEn. But neither FuzzEn nor SampEn was capable of differentiating the Logistic attractors with $\mu = 4.0$ from those with $\mu = 3.5$, once the percentage of additive noise was greater than 35%. However, the rFuzzyEn showed very small standard deviations (see the range marked by gray bar in Figure 3). It could well differentiate between the two Logistic attractors even when the percentage of additive noise increased to 60%.

## Experimental results

**Figure 4:**
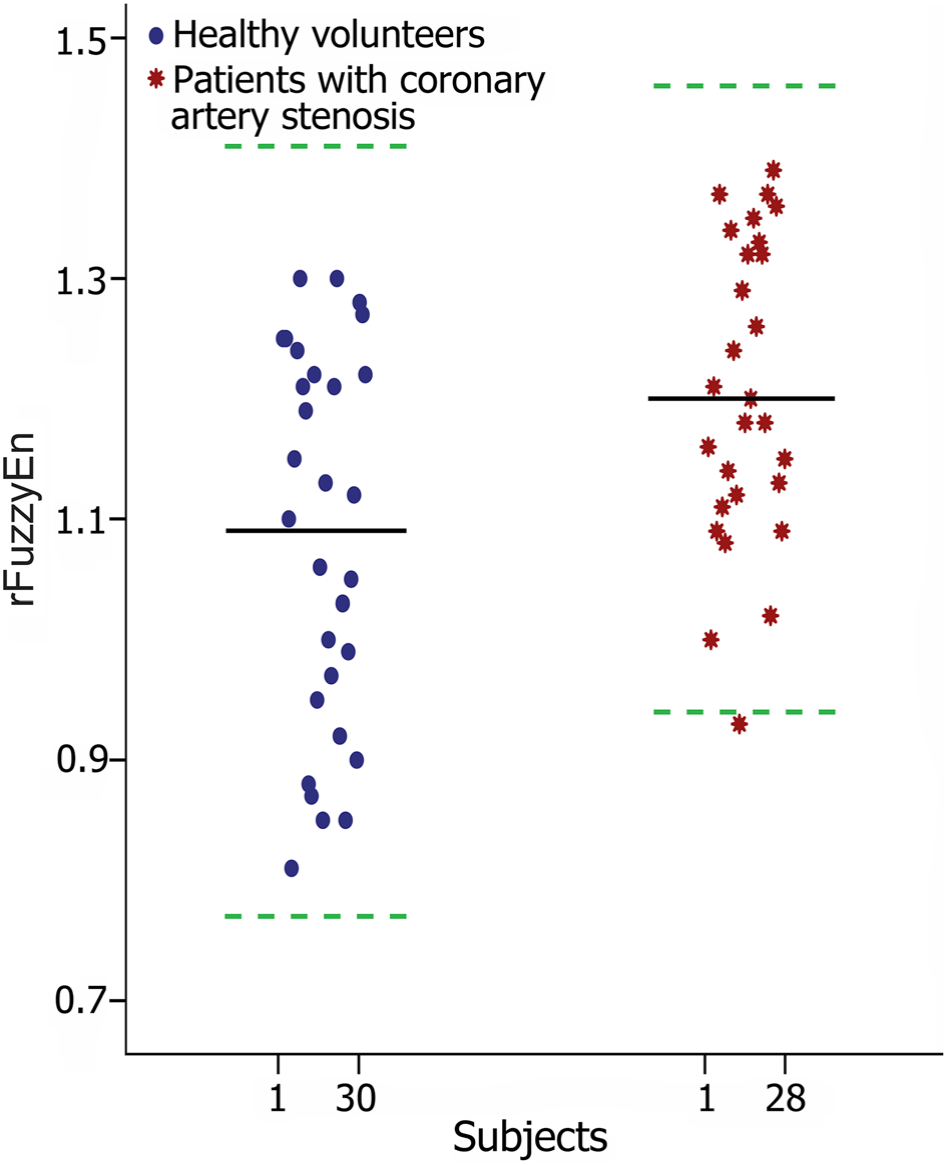
The Bland–Altman plot of rFuzzyEn of DPV for all subjects in the two groups. The *black solid line* indicates the mean and the *green dotted line* indicates the *upper* and *lower* bounds of the mean ± 2 SD, respectively.

Table 2 shows the linear (time- and frequency- domain) and nonlinear indices of HRV, and the corresponding DPV results are shown in Table 3. Statistical analyses showed that all HRV indices, including the rFuzzyEn measure, did not have significant differences between patients with coronary artery stenosis and healthy volunteers (all *p* > 0.1). Neither the linear (SD, LF, HF) nor the nonlinear SampEn indices of DPV showed significant difference between the two groups (all *p* > 0.1). The FuzzyEn increased in patients with coronary artery stenosis but the difference was less statistically significant (*p* = 0.05) compared with the healthy volunteers. However, results showed a statistically significant increase in the rFuzzyEn for patients with coronary artery stenosis (*p* < 0.01). To further illustrate the performance of rFuzzyEn in analysing the DPV, we show its Bland–Altman plot in Figure 4. The mean rFuzzyEn increased from 1.09 in healthy volunteers to 1.20 in patients with coronary artery stenosis.

**Table 2:** The time/frequency domain and entropy features of HRV

Variables	Healthy volunteers	Patients with coronary artery stenosis	*p*
SD (ms)	28.00 ± 12.91	25.81 ± 8.64	0.45
LF (ms^2^)	97.51 [66.71 185.33]	77.74 [50.93 127.13]	0.10
HF (ms^2^)	114.72 [52.76 169.30]	114.55 [51.64 170.06]	0.98
SampEn	1.92 ± 0.33	1.95 ± 0.35	0.79
FuzzyEn	1.49 ± 0.22	1.53 ± 0.20	0.44
rFuzzyEn	1.21 ± 0.14	1.25 ± 0.11	0.21

*SD* standard deviation, *LF* power of low-frequency band, *HF* power of high-frequency band, *SampEn* sample entropy, *FuzzyEn* fuzzy entropy, *rFuzzyEn* refined fuzzy entropy. Data are expressed as mean ± standard deviation (SD) or median [25% 75%].

**Table 3:** The time/frequency domain and entropy features of DPV

Variables	Healthy volunteers	Patients with coronary artery stenosis	*p*
SD (ms)	29.86 ± 13.46	26.32 ± 8.94	0.25
LF (ms^2^)	121.04 [86.20 213.86]	91.09 [59.53 188.22]	0.14
HF (ms^2^)	86.42 [37.86 131.04]	53.87 [29.89 128.03]	0.19
SampEn	1.93 ± 0.44	2.08 ± 0.41	0.14
FuzzyEn	1.42 ± 0.27	1.54 ± 0.23	0.05
rFuzzyEn	1.09 ± 0.16	1.20 ± 0.13	<0.01

*SD* standard deviation, *LF* power of low-frequency band, *HF* power of high-frequency band, *SampEn* sample entropy, *FuzzyEn* fuzzy entropy, *rFuzzyEn* refined fuzzy entropy. Data are expressed as mean ± standard deviation (SD) or median [25% 75%].

## Discussion

In this study, we developed a refined fuzzy entropy (rFuzzyEn) measure by substituting a piecewise fuzzy membership function for the Gaussian function in the traditional FuzzyEn measure. Its stability and robustness against the additive noise were examined by two well-studied synthetic models—the $1/f^{\alpha }$ noise and Logistic attractors, in comparison with the SampEn and FuzzyEn measures. Simulation results indicated that the rFuzzyEn outperformed the traditional SampEn and FuzzyEn measures in terms of both stability and robustness against additive noise, particularly with small data set. Results from the simulation tests suggest that the rFuzzyEn is more suited for analysing short-term series than traditional SampEn and FuzzyEn measures.

The rFuzzyEn has subsequently been applied to analyse clinical short-term HRV and DPV series recorded from the patients with coronary artery stenosis and healthy volunteers. No significant difference was found between the two groups in time/frequency-domain indices of both HRV and DPV, which again suggests that those linear time-invariant approaches are not suited for short-term physiological series analyses. In addition, experimental results also showed that none of the three measures (SampEn, FuzzyEn, and rFuzzyEn) could detect an effect of coronary artery stenosis on the HRV, which, however, differs from previous findings [2, 11]. Since Carney et al. reported that certain psychological conditions in patients with coronary artery stenosis, such as the major depression, had great influence on their HRV [34], the mentioned difference might be partly due to that we did not take those psychological conditions into consideration. Data sets might be another factor, that accounts for this discrepancy. The current study focused on the short-term HRV series, which should be different from the long-term HRV series analysed in the previous studies [11]. For the DPV analysis, however, even without significant difference being found between the two groups in SampEn, a mild increase of FuzzyEn and a more remarkable increase of rFuzzyEn were observed in patients with coronary artery stenosis. Since DPV contributes most to HRV physiologically, whereas the beat-to-beat systolic intervals varies less so as to support a relatively stable stroke volume, cardiac output, and steady-going blood supplies [13, 14], the increased FuzzyEn and rFuzzyEn of DPV might suggest abnormal beat-to-beat systolic intervals fluctuations in patients with coronary artery stenosis which leads to irregularity in the DPV series. The short-term DPV analysis may thus provide valuable information in addition to HRV.

The Bland–Altman plot in Figure 4 further suggested that although the difference in rFuzzyEn was statistically significant, it was likely to be of minor clinical meaning because the difference was in fact very tiny (from 1.09 increased to 1.20). The sensitivity and specificity in discriminating the two groups by only rFuzzyEn would not be large enough to be accepted clinically. However, it should be promising to work as an input feature for specific classification task.

In addition, only one test among all the 19 statistical tests (Tables 1, 2, 3) showed statistically significant. This might happen by chance. We thus used the Bonferroni correction method to make further examination (considering all the DPV parameters as one family and the statistical level reduced to be 0.05 divided by 6, that is around 0.008) [35]. The rFuzzyEn of DPV in patients with coronary artery stenosis still showed a statistically significant increase (*p* = 0.006).

Moreover, according to both the simulation and experimental results, it still leaves room for improvement of rFuzzyEn. For example, although rFuzzyEn could distinguish the Logistic attractor with $\mu = 3.5$ from that with $\mu = 4.0$ (Figure 3), the difference between the two rFuzzyEn results was tiny. Recently, Li et al. have proved that the thresholding process in the calculation framework of traditional SampEn-based measures accounts most for the poor performances in short-term series analysis. They have developed a new measure—distribution entropy—by removing the thresholding process, and have shown its advantages in analysis of extremely small data set over the SampEn and FuzzyEn [36]. The distribution entropy has a potential in short-term HRV and DPV analysis. We will investigate its performance in our future studies.

## Conclusions

A piecewise fuzzy membership function-based rFuzzyEn measure has been developed in this study. It showed improved stability and robustness against additive noise in the simulation tests. Its performance was subsequently validated by clinical short-term HRV and DPV series. The experimental results further suggested that the patients with coronary artery stenosis exhibit a significantly elevation in the rFuzzyEn of short-term DPV series, whereas no statistically significant difference was found in the rFuzzyEn of HRV series. Improved performance in recognition of those patients through DPV analysis should also be expected. Studies on the non-invasive evaluation of coronary artery stenosis may benefit from the above results.
